# Amyloid β induces interneuron-specific changes in the hippocampus of APP^NL-F^ mice

**DOI:** 10.1371/journal.pone.0233700

**Published:** 2020-05-29

**Authors:** Katalin E. Sos, Márton I. Mayer, Virág T. Takács, Abel Major, Zsuzsanna Bardóczi, Barnabas M. Beres, Tamás Szeles, Takashi Saito, Takaomi C. Saido, István Mody, Tamás F. Freund, Gábor Nyiri

**Affiliations:** 1 Department of Cellular and Network Neurobiology, Institute of Experimental Medicine, HAS, Budapest, Hungary; 2 János Szentágothai Doctoral School of Neurosciences, Semmelweis University, Budapest, Hungary; 3 Laboratory for Proteolytic Neuroscience, RIKEN, Center for Brain Science, Saitama, Japan; 4 Department of Neurocognitive Science, Nagoya City University Graduate School of Medical Science, Aichi, Japan; 5 Department of Neurology, University of California, Los Angeles, California, United States of America; Sungkyunkwan University, REPUBLIC OF KOREA

## Abstract

Alzheimer’s disease (AD) is a neurodegenerative disorder characterized by cognitive decline and amyloid-beta (Aβ) depositions generated by the proteolysis of amyloid precursor protein (APP) in the brain. In APP^NL-F^ mice, APP gene was humanized and contains two familial AD mutations, and APP–unlike other mouse models of AD–is driven by the endogenous mouse APP promoter. Similar to people without apparent cognitive dysfunction but with heavy Aβ plaque load, we found no significant decline in the working memory of adult APP^NL-F^ mice, but these mice showed decline in the expression of normal anxiety. Using immunohistochemistry and 3D block-face scanning electron microscopy, we found no changes in GABA_A_ receptor positivity and size of somatic and dendritic synapses of hippocampal interneurons. We did not find alterations in the level of expression of perineuronal nets around parvalbumin (PV) interneurons or in the density of PV- or somatostatin-positive hippocampal interneurons. However, in contrast to other investigated cell types, PV interneuron axons were occasionally mildly dystrophic around Aβ plaques, and the synapses of PV-positive axon initial segment (AIS)-targeting interneurons were significantly enlarged. Our results suggest that PV interneurons are highly resistant to amyloidosis in APP^NL-F^ mice and amyloid-induced increase in hippocampal pyramidal cell excitability may be compensated by PV-positive AIS-targeting cells. Mechanisms that make PV neurons more resilient could therefore be exploited in the treatment of AD for mitigating Aβ-related inflammatory effects on neurons.

## Introduction

Dementia is a progressive multifactorial disorder influenced by genetic and environmental factors [1–4]. Alzheimer’s disease (AD) is the most common type of dementia with neurodegeneration and cognitive decline accompanied by depositions of amyloid-beta (Aβ) protein (extracellular amyloid plaques) and intracellular tangles of axonal protein Tau in the brain [5,6]. For decades, it was hypothesized that the primary trigger for the pathogenesis of AD was the accumulation of Aβ [7–9] that was also responsible for Tau-pathology [5]. Numerous studies have proposed that removal of Aβ could prevent the disease [10–13]. Indeed, removal of Aβ from the brains of transgenic mice with increased levels of Aβ was associated with behavioral improvements [14–16]. Currently, several clinical trials aim to eliminate accumulated Aβ from patient’s brains [10,17,18], however, most of these clinical efforts have failed [11,12,19]. In addition, some postmortem brains of people who have died in old age without apparent cognitive dysfunction show at least as heavy a plaque load as brains from patients with advanced symptoms of AD [5,20–24]. These apparent discrepancies make it especially important to better understand both the natural course of Aβ accumulation and the mouse models used for AD research.

Although all forms of AD seem to involve a rise in Aβ levels, the causes of the elevated Aβ may be diverse. In familial AD, genetic mutations in the synthetic pathway of Aβ generate higher concentrations of Aβ or alter the relative levels of different Aβ isoforms [9,25,26]. In sporadic AD, several factors may contribute to the causes including inflammation, type 2 diabetes, obesity, head trauma, ischemia or other environmental factors, possibly with a combination of some genetic factors. Aβ is generated by the proteolysis of amyloid precursor protein (APP), certain mutations of which lead to the accumulation of Aβ plaques in the brain [8]. Once plaque formation starts, their size can increase by attracting further Aβ deposition [27,28]. In most mouse models of AD and postmortem human AD tissue, amyloid plaques are surrounded by thickened, irregular neuronal processes called dystrophic neurites (DN) [29–31]considered to be a response to inflammation induced by the neurotoxic soluble oligomeric Aβ [32]. Amyloid plaques and DN are present in cortical areas, but they are highly abundant in the hippocampus [33,34] and are generally surrounded by activated glial processes [29,35–37] that try to rescue surrounding tissue from further damage. Several microglial genes are associated with the increased risk of AD, suggesting that these brain-resident immune cells play a role in disease progression or natural prevention [38–40].

In hippocampus, GABAergic interneurons (INs) synchronously inhibit different membrane domains of pyramidal cells and have a crucial role in memory formation [41–46]. Parvalbumin (PV)-positive IN terminals mainly inhibit somatic membranes or axon initial segments (AIS) of pyramidal cells, whereas most local somatostatin (SOM)-positive INs target distal dendrites of pyramidal cells [45–50]. Previous studies suggested that several populations of INs, including PV and SOM INs, are highly sensitive for Aβ accumulation [51–56] and loss of function of these INs may be responsible for hyperexcitability [57] or epileptic activity in AD [58]. However it is unclear whether any of these observations could be artefacts because of the unnatural overexpression of APP in those mouse models [59].

More than one hundred mouse models were developed for investigating the effects of Aβ accumulation. Expression of their mutated APP was controlled by an artificially strong non-specific promoter that forced the overexpression of APP even in populations of cells that would not necessarily express APP, especially not in a large quantity [60,61]. Overexpression could generate effects in neurons that otherwise would have never occurred in AD [62]. Furthermore, these mice also show an increased level of the proteolytical fragments of APP that may induce Aβ-independent artefacts [63]. To mitigate these issues, here we used APP^NL-F^ mice, which express APP driven by its endogenous mouse promoter [64]. To closely mimic human phenotype, the APP^NL-F^ mouse APP gene was first humanized and then it was modified with a Swedish and an Iberian familial AD mutation [65]. APP^NL-F^ mice also closely mimic the critical ratio of Aβ isoforms found in human patients [64]. Previous behavioral experiments showed modest abnormalities in APP^NL-F^ mice most often only beyond the age of one year. Older APP^NL-F^ mice seem to be less effective in novel object recognition [66], working memory [64], fear learning [66], place avoidance learning and aversive memory tasks [67]. However, some of these tasks are also challenging for aging wild type (WT) mice [59,67].

Here we investigated morphological alterations, synaptic GABA_A_ receptor content, synaptic areas, changes in hippocampal interneuronal connections and numbers, and behavioral alterations in APP^NL-F^ mice. We found that most investigated parameters were not different in APP^NL-F^ mice. However, we found that the area of synapses of axon initial segment (AIS)-targeting axo-axonic INs were enlarged significantly. Contrary to data from other models, we found that PV INs are particularly resistant to amyloidosis in APP^NL-F^ mice. The expression of perineuronal nets around PV-cells seemed to be unaffected and APP^NL-F^ mice did not show a significant difference in the density of PV and SOM positive INs. In addition, PV-positive axonal fibers were rarely dystrophic.

Our results showed that even at old age APP^NL-F^ mice display only mild anatomical signs and behavioral symptoms. This suggests that even massive amyloid plaque-formations can have relatively moderate effects alone on neural network function, similar to patients with high Aβ plaque density without cognitive impairment [5,20–24], which may be due to the effective protection provided by glial cells [5,68,69]. However, our results also suggest that amyloid-induced increase in excitatory activity [28,70] may change the inhibitory balance in hippocampus. This is realized by increasing a compensatory inhibition of pyramidal cells on their AIS by PV-positive axo-axonic cells that seem to be resistant to Aβ.

## Materials and methods

### Animals

All experiments were performed in accordance with the Institutional Ethical Codex, Hungarian Act of Animal Care and Experimentation (1998, XXVIII, section 243/1998) and the European Union guidelines (directive 2010/63/EU), and with the approval of the Institutional Animal Care and Use Committee of the Institute of Experimental Medicine of the Hungarian Academy of Sciences. Experiments were approved under the project number PE/EA/665-2/2017. All efforts were made to minimize potential pain or suffering and to reduce the number of animals used.

APP^NL-F^ mice were derived from the Riken Institute colony (Japan). Mice carry Swedish mutation that elevates the total amount of Aβ40 and Aβ42, and Iberian mutation that increases the ratio of Aβ42 to Aβ40 (APP^NL-F^ mice) [64]. The numbers and ages of APP^NL-F^ mice and WT littermates used in this study are indicated below for each experiment. APP^NL-F^ mice were crossbred with transgenic mice expressing enhanced green fluorescent protein (eGFP) controlled by PV promoter (BAC-PV-eGFP) to facilitate stereology-based investigations of PV neurons. APP^NL-F^ mice were crossbreed with Pvalb-IRES-Cre (The Jackson Laboratory; RRID: IMSR_JAX: 008069) to better understand the effect of plaques on septo-hippocampal PV fibers.

### Surgery

Mice were shortly anesthetized with isoflurane immediately followed by an intraperitoneal injection of an anesthetic mixture (containing 8.3 mg/ml ketamine, 1.7 mg/ml xylazine-hydrochloride in 0.9% saline, 10 ml/kg body weight) and then were mounted in a stereotaxic frame. For selective labeling of hippocampal PV positive INs we injected 2x60nl AAV2/5-EF1a-DIO-mCherry virus into one-side of the dorsal hippocampus, coordinates: Br-1.8, y-1.2, z-1.8 and Br-2.4, y-2.0, z-1.9. For selective labeling of septo-hippocampal PV positive axons, we injected 30 nl AAV2/5-EF1a-DIO-eYFP tracer virus (UNC Vector Core) into the medial septal area (1.0 mm anterior from the bregma, in the midline, and -4.3 mm below the level of the horizontal plane defined by the bregma and the lambda (zero level). The coordinates for the injection were defined based on the atlas by Paxinos [71]. For the injections, we used a Nanoject 2010 precision micro-injector pump (WPI, Sarasota, FL 34240). We used borosilicate micropipettes (Drummond, Broomall, PA) for the injections with tips broken to 40–50 μm. After the surgeries, animals received 0.5–0.7 ml saline for rehydration and 0.03–0.05 mg/kg meloxicam as non-steroidal anti-inflammatory medication (Metacam, Boehringer Ingelheim, Germany) intraperitoneally to support recovery, and we placed them into separate cages to survive for at least three weeks.

### Perfusions

Mice were anesthetized with isoflurane, followed by intraperitoneal injection of an anesthetic mixture (containing 8.3 mg/ml ketamine, 1.7 mg/ml xylazine-hydrochloride, 0.8 mg/ml promethazinium-chloride) to achieve deep anesthesia. Then, mice were perfused transcardially first with phosphate-buffered saline solution (PBS, 0.9% NaCl in 0.1M phosphate buffer) for 2 min, followed by 4% paraformaldehyde (PFA) for 40 min and finally with 0.1 M phosphate buffer (pH: 7.4, PB) for 10 min. Mice for electron microscopy detection of GABA_A_ receptors were perfused transcardially with ice-cold, oxygenated artificial cerebrospinal fluid [containing (mM) NaCl 125, KCl 2.5, CaCl_2_ 2.5, MgCl_2_ 2, NaHCO_3_ 26, NaH_2_PO_4_ 1.25, glucose 25]. Then the brain was removed from the skull and cut in blocks containing the hippocampal formation. The blocks were incubated in a fixative containing 4% PFA and 0.2% glutaraldehyde in PB for 120 min at room temperature and then embedded in 2% agarose for sectioning. Coronal sections were cut on a vibratome (Leica VT1200S) at 50 or 60 μm. Animals for block-face scanning electron microscopy (BF-SEM) microscopy were anaesthetized with isoflurane and then were decapitated. The brains were removed from the skull on ice, cut sagittally and coronally into 6 pieces and immersion fixed in 2% PFA, 1% glutaraldehyde and 15% saturated picric acid solution in 0.1 M phosphate buffer (PB) for 2 hours at room temperature, then the fixative was washed out with 0.1 M PB. Brain pieces containing hippocampi were sectioned with a vibratome (Leica VT1200S) to 150 μm thickness.

### Antibodies

We summarized data for antibodies in Tables 1 and 2. The secondary antibodies were also extensively tested for possible cross-reactivity with the other secondary or primary antibodies, and possible tissue labeling without primary antibodies was also tested to exclude autofluorescence or specific background labeling by the secondary antibodies. No specific staining was observed under these control conditions.

**Table 1:** Primary antibodies.

Raised against	Raised in	Dilution* (application)	Source	Catalog number/ code	Specificity
**Amyloid-beta (MOAB-2)**	mouse monoclonal	1:500	Abcam	ab126649	Monoclonal antibody detects unaggregated, oligomeric and fibrillar forms of Aß42, and unaggregated Aß40. Does not detect APP.
**IBA1**	rabbit	1:1000	FUJIFILM Wako Pure Chemical Corporation	01919741	Specific to microglia and macrophage, but not cross-reactive with neuron and astrocyte.
**GFAP (Gliall-specific type-III intermediate filament protein**	chicken	1:1000	Synaptic Systems	173006	Recombinant protein corresponding to AA 1 to 432 from human GFAP (UniProt Id: P14136)
**Green fluorescent protein (GFP)**	chicken	1:2000 (DAB, fluorescent)	Molecular Probes	A10262	No labeling in animals that were not injected with eGFP-expressing viruses
**mCherry**	Rabbit	1:2000	BioVision	5993–100	
**Biotinylated Wisteria Floribunda Lectin (PNN)**	lectin	1:5000	Vector Laboratories	B-1355, lot: Y0409	Preferentially bind carbohydrate structures terminating in GalNAc linked α or β to the 3 or 6 position of galactose. (manufacturer’s information)
**Vesicular acetylcholine transporter (vAChT)**	goat	1:10000(DAB, fluorescent)	Immunostar	1308002	Immunolabeling is completely abolished by preadsorption with synthetic rat VAT (vAChT) (511–530). (manufacturer’s information)
**GABA**_**A**_ **receptor gamma 2 subunit**	rabbit	1:1000 (preembedding immunogold)	Synaptic Systems	224 003	Immunolabeling is abolished by virus-mediated gene-knock-out of GABA_A_ receptors (Rovó et al. 2014)
**PV**	rabbit	1:2000 (fluorescent)	SWANT	235	KO verified (manufacturer’s information)
**PV**	mouse	1:1000 (fluorescent)	Dr. Kenneth Baimbridge	-	stains the same as the KO-verified mouse ab
**TH**	Mouse	1:2000	ImmunoStar	22941	
**vGAT**	guinea pig	1:2000	Synaptic Systems	131 004	
**VGLUT1**	guinea pig	1:1000	Millipore	AB5905	
**CB1 receptor**	rabbit	1:1000	Cayman Chemicals	10006590	Synthetic peptide from the C-terminal region of human protein CB1 receptor
**Somatostatin**	guinea pig	1:500 (fluorescent, DAB)	Synaptic Systems	366004	This antibody preferentially recognizes somatostatin-28.

**Table 2:** Secondary antibodies.

Conjugated with	Raised in	Raised against	Dilution* (procedure)	Source	Catalog number
**1.4-nm nanogold**	Goat	rabbit	1:100–1:300 (preembedding immunogold)	Nanoprobes	#2004
**Ultra small gold**	Goat	mouse	1:50 (preembedding immunogold)	Aurion	100.022
**Biotin-SP**	donkey	goat	1:1000 (DAB)	JIL Inc. (Jackson Immunoresearch Laboratories, Inc.)	705-066-147
**Biotin-SP**	donkey	mouse	1:1000 (DAB)	JIL Inc.	715-066-151
**Biotin-SP**	donkey	goat	1:1000 (DAB)	JIL Inc.	705-066-147
**Biotin-SP**	donkey	rabbit	1:500 (DAB)	JIL Inc.	711-065-152
**Biotin**	donkey	guinea-pig	1:500 (DAB)	Vector Laboratories	BA-7000
**Alexa 488**	Goat	chicken	1:1000 (confocal)	Vector Laboratories	A-11039
**Alexa 488**	donkey	chicken	1:1000 (confocal)	JIL Inc.	703-545-155
**Alexa 594**	donkey	rabbit	1:500 (confocal)	Invitrogen	A21207
**Alexa 647**	donkey	mouse	1:500 (confocal)	JIL Inc.	715-605-151
**Alexa 594**		Streptavidin	1:500 (confocal)	Molecular Probes	S32356

### Immunofluorescent labeling and confocal laser-scanning microscopy

Before immunofluorescent staining, 50 μm thick sections were washed in PB and Tris-buffered saline (TBS). This was followed by blocking for 1 hour in 1% human serum albumin (HSA; Sigma-Aldrich) and 0.1% Triton X-100 (and 1 mg/ml Digitonin in case of amyloid beta antibody) dissolved in TBS. After this, the sections were incubated in mixtures of primary antibodies (Table 1) overnight at room temperature. Then sections were washed in TBS and were incubated overnight at 4 °C in a mixture of secondary antibodies (Table 2), all diluted in TBS, followed by washes in TBS and PB. Then sections were mounted on glass slides, and cover-slipped with Aqua-Mount (Polysciences). Immunofluorescence was analyzed using a Nikon Eclipse Ti-E inverted microscope (Nikon Instruments Europe B.V., Amsterdam, The Netherlands) and an A1R laser confocal system. We used 488, 561 and 642 nm lasers (CVI Melles Griot), and scanning was done in line serial mode. Image stacks were obtained with NIS-Elements AR 4.5 software.

### Single and double-labeling preembedding immunoelectron microscopy

Sections were freeze-thawed three times over liquid nitrogen and washed in PB. For detection of GABA_A_ receptors, sections were incubated in 0.2 M HCl solution containing 2 mg/ml pepsin (Dako) at 37 °C for 2–4 min. After extensive washes in PB and 0.05 M TBS (pH 7.4) sections were blocked in 1% human serum albumin (HSA; Sigma-Aldrich) in TBS. Then, they were incubated in a mixture of primary antibodies diluted in TBS containing 0.05% sodium azide for 2–3 days at 4 °C in fridge. After repeated washes in TBS, sections were incubated in blocking solution (Gel-BS) containing 0.2% cold water fish skin gelatin and 0.5% HSA in TBS for 1 h. Then, sections were incubated in gold-conjugated and biotinylated secondary antibodies diluted in Gel-BS overnight. After extensive washes in TBS, the sections were treated with 2% glutaraldehyde in 0.1 M PB for 15 min to fix the gold particles into the tissue. This was followed by incubation in avidin–biotinylated horseradish peroxidase complex (Elite ABC; 1:300; Vector Laboratories) diluted in TBS for 3 h at room temperature or overnight at 4 °C. The immunoperoxidase reaction was developed using 3,3-diaminobenzidine (DAB; Sigma-Aldrich) as chromogen. To enlarge immunogold particles, sections were incubated in silver enhancement solution (SE-EM; Aurion) for 40 min at room temperature. The sections were then treated with 0.5% OsO_4_ in 0.1 M PB on ice, dehydrated in ascending alcohol series and in acetonitrile and embedded in Durcupan (ACM; Fluka). During dehydration, the sections were treated with 1% uranyl acetate in 70% ethanol for 20 min. For electron microscopic analysis, tissue samples from the CA1 area of dorsal hippocampus were glued onto Durcupan blocks. Consecutive 60 nm-thick sections were cut using an ultramicrotome (Leica EM UC6) and picked up on Formvar-coated single-slot grids (for conventional electron microscopic analysis). Ultrathin sections for conventional electron microscopic analysis were counterstained with lead citrate (Ultrostain 2, Leica) and examined in a Hitachi 7100 electron microscope equipped with a Veleta CCD camera (Olympus Soft Imaging Solutions, Germany).

### Cell density estimations

In 60 μm thick sections images were taken using a Nikon Ni-E Eclipse light microscope with a 10x objective. Image stacks were recorded the whole thickness of the sections at a z-distance of 5.6 μm with a Nikon DS-Fi3 camera. Pixel size was 0.57 μm. Measurements were done with stereology-based counting on 8-bit, grayscale photos using NIS-Elements AR 4.5 software. First, the hippocampal areas were defined by delineating them, and were established by the software. During measurements, we counted all PV INs in systematic randomly-selected sections in CA1-3 and dentate gyrus. SOM INs were also counted in systematic randomly-selected sections in CA1 stratum oriens. Then, data were exported into Microsoft Excel. Because some counted cells were cut at the surface of the sections we used Abercrombie correction [72] to correct for potential oversampling. For the correction, images were taken using a Zeiss Axioskop 2 mot plus light microscopy using 40x objective and a Retiga 2000R camera. The measurement of section thickness and cell diameter was done with random sampling in the area of the hippocampus. The thickness was measured at five points in each section and averaged. Cellular diameter was calculated from the average of 30 cells in each section. The values of the fractions were 0.78 for both APP^NL-F^ and WT mice. The results indicated that both of the parameters showed negligible difference in each animal, but still, corrections were applied. Every tenth section were used for cell density estimation.

### Electron microscopy

For evaluation of the ratio of GABA_A_ receptor positive synapses of cholinergic, SOM- and PV-positive terminals, we performed preembedding double labeling. Vesicular acetylcholine transporter (vAChT), SOM and PV were labeled with DAB, while GABA_A_ receptor γ2 subunits were labeled with immunogold. Electron microscopic serial sections were systematically scanned for synapses of DAB-labeled terminals. Synapses were followed and photographed at 30,000 times magnification. Because background labeling (measured in putative glutamatergic synapses in the same series of sections) was negligible, synapses containing at least one gold particle were regarded as positive.

### Block-face scanning electron microscopy (BF-SEM)

For BF-SEM we used a published protocol [73]. Sections were post-fixed in 1% osmium-tetroxide reduced with 0.75% potassium ferrocyanide, on ice for 1 hour, followed by thiocarbohydrazide (TCH) incubation for 30 minutes. Then sections were treated with 1% osmium-tetroxide solution for 30 minutes, then for another 30 minutes with 1% aqueous uranyl-acetate in dark. Then, Walton’s *en bloc* lead aspartate staining was performed at 60°C for 30 minutes. After each step, 5 x 3 minutes distilled water washes were performed. Finally, the sections were dehydrated through ascending concentration of ethanol series on ice (from 30% to absolute ethanol, each step took 2x8 minutes) and then infiltrated with acetonitrile 2 x 10 minutes (first on ice, second at room temperature). The sections were transferred into aluminum boats and infiltrated with embedding resin (Epoxy Embedding Medium Kit, Sigma-Aldrich, hard mixture) overnight. On the next day, sections were mounted on glass slides and covered with Aclar sheets (Electron Microscopy Sciences) and baked at 60°C for 48 hours. After that, a small region of interest, in the CA1 region of the hippocampus was cut out with a razor blade and mounted on an aluminum specimen pin with Silver Conductive Epoxy, H2OE EPO-TEK^®^ (Ted Pella) and baked at 60°C for 48 hours/ 30 minutes at 120°C. After consolidation of the conductive epoxy, the samples were trimmed with an ultracut (EM UC6, Leica, Wetzlar, Germany) with a glass knife into a cube (~ 400 μm x 400 μm x 75 μm) and were sputter coated with gold by Rotary-Pumped Sputter Coater (Quorum Technologies, Q150R ES). The samples were stored in dust free holders and before using for BF-SEM imaging they were transferred into the vacuum chamber for at least one night.

### BF-SEM imaging

We used a FEI Apreo SEM with field emission gun equipped with an in situ ultramicrotome (VolumeScope, FEI, Eindhoven, The Netherlands) and a T1 “in-column” detector to record the backscattered electrons (BSE) from each voxel. For all image stacks, we collected BSE images with MAPs software (FEI) from 6.5 mm working distance at high vacuum with fixed 0.1 nA beam current. For imaging AISs, basket cells and SOM Ins, we recorded image stacks with the following parameters: 2.0 kV high voltage, 1.20 μs/pixel dwell time, and 4 nm x 4 nm x 70 nm voxel size. The micrographs were 16384x16383 pixels, and we recorded 300 slices with 70 nm thickness, therefore the overall volume for each animal was cca. 91 200 μm^3^. For the dystrophic neurites, pixel size was 9 nm and the slice thickness was 280 nm across 110 slices.

### Image analysis

SEM image post-processing was done in Fiji ImageJ [74]. SEM micrographs were inverted, de-noised and sharpened to enhance image quality. The BF-SEM stacks were imported into TrakEM2 [75] and were fine aligned. For segmentations, delineation was performed on a Wacom Cintiq 27QHD Creative Pen and display tablet. We used area lists for axonal profiles, mitochondria, and profile lists for synapses. Semi-manual segmentation was carried out, for axon profiles on every 2nd or 3rd slices and we used the built-in interpolation method. After, synaptic surface area, mitochondria volume, AIS length measurements were finished using the built-in plugin of TrakEM2, data were retrieved by custom made excel datasheet and analyzed with Tibco Statistica 13.4. After segmentation, the models were exported in “.obj” file format and imported into Blender (Blender Foundation, Amsterdam, The Netherlands) for further investigation and visualization.

### Spontaneous alternation test

The working memory of APP^NL-F^ mice and WT littermates was tested in spontaneous alternation test paradigm in a 4 arm plus maze, where each arm was 30 cm x 7 cm with 30-cm-high walls. Before these experiments, mice did not perform in any other tests. Each mouse was placed in the center of the plus maze and allowed to explore freely during a ten-minute test period. Mice with good working memory can explore all arms efficiently, visiting arms one after the other without revisiting them too often. As the mouse moves through each arm, optimally it should remember which arm was visited and try to enter a different one. To analyze this behavior, the sequence and total amount of arm entries were recorded and a percentage was calculated for perfect alternation (visiting all four arms one after another, e.g. A-B-C-D) and for mistakes of different types (A-B-C-A, A-B-A, A-A). All limbs of the mouse had to be located on the same arm in order to record an arm entry. Experimental data were collected and analyzed using Noldus EthoVision XT 12 and Tibco Statistica 13 software.

### Elevated Plus Maze (EPM)

Animals were placed into an EPM to measure anxiety levels. Cross-shaped EPM apparatus consisted of two opposite open arms (30 cm x 7 cm) without walls and two closed arms with 30-cm-high walls, on a platform 50 cm above floor level. Each mouse was placed in the center of the EPM and was allowed to explore freely during a five-minute test period. The total time spent in the open arms was measured. All limbs of the mouse had to be located on the same arm in order to detect the mouse in that arm. Experimental data were collected and analyzed using Noldus EthoVision XT 12 and Tibco Statistica 13 software.

### Trace fear conditioning

Animals were handled for 3 days, then habituated to the experimental box (with every environmental cue of the first experimental day) for 2 days. Behavioral experiments were performed in a separate experimental room. On day 6, mice were placed first into environment “A” in a plexiglass shocking chamber (25 cm x 25 cm x 31 cm) that was enriched with a specific combination of olfactory (baby soap scent), visual (dotty walls), spatial (bended chamber wall), auditory (constant recorded noise sound) and tactile (stainless steel bars on the floor) cues. Animals could freely move in the first environment for 3 minutes to record baseline freezing levels. Then, mice received 3 repeats of the following cycle: 20 sec auditory cue and after 18 sec they received a 2 sec, 2mA footshock followed by 3 min inter-cue interval. After 3 successful shocks, animals were placed back into their home cages for 24 hours. On day 7, mice were placed into environment “B” which consisted of another set of olfactory (macadamia nut scent), visual (striped wall), spatial (rectangle shape chamber), auditory (no noise) and tactile (plastic floor under their feet) cues. To read out cued fear (freezing) behavior, mice could freely move in environment “B” for 3 min (baseline), then they received 3 repeats of the following cycle: 60 sec auditory cue, 3 min readout. On day 8, animals were placed back into environment “A” for 3 min for contextual fear readout. Each training chamber was cleaned using the same scented liquid hand wash soap before each mouse. The behavior of mice was recorded with a Basler camera (Basler AG, Ahrensburg, Germany), and freezing behavior was analyzed using an automated system (Noldus Ethovision 12.0; Noldus Interactive Technologies). Mice were considered to show freezing behavior, if they did not show any movement, other than breathing, for at least 2 seconds. Animals with baseline freezing higher than 5% were excluded from further analysis. The experimenter evaluating the freezing levels was blind to the conditions of the animals.

### Statistics

In case of data groups that did not display a Gaussian distribution, we used median and 25%-75% interquartile range to describe data. We used means and standard deviations to describe data groups that displayed Gaussian distribution. Gaussian distribution was tested using Shapiro-Wilk test. To test for statistical differences, we used the non-parametric Mann-Whitney U-test or parametric Student’s t-test in independent data populations. Statistical difference have always been tested using two-sided tests. Homogeneity of variance was tested using F-test and if it was significant then populations were compared using nonparametric tests.

## Results

### Dystrophic neurites (DN) and surrounding glia are abundant in APP^NL-F^ mice

**Fig 1:**
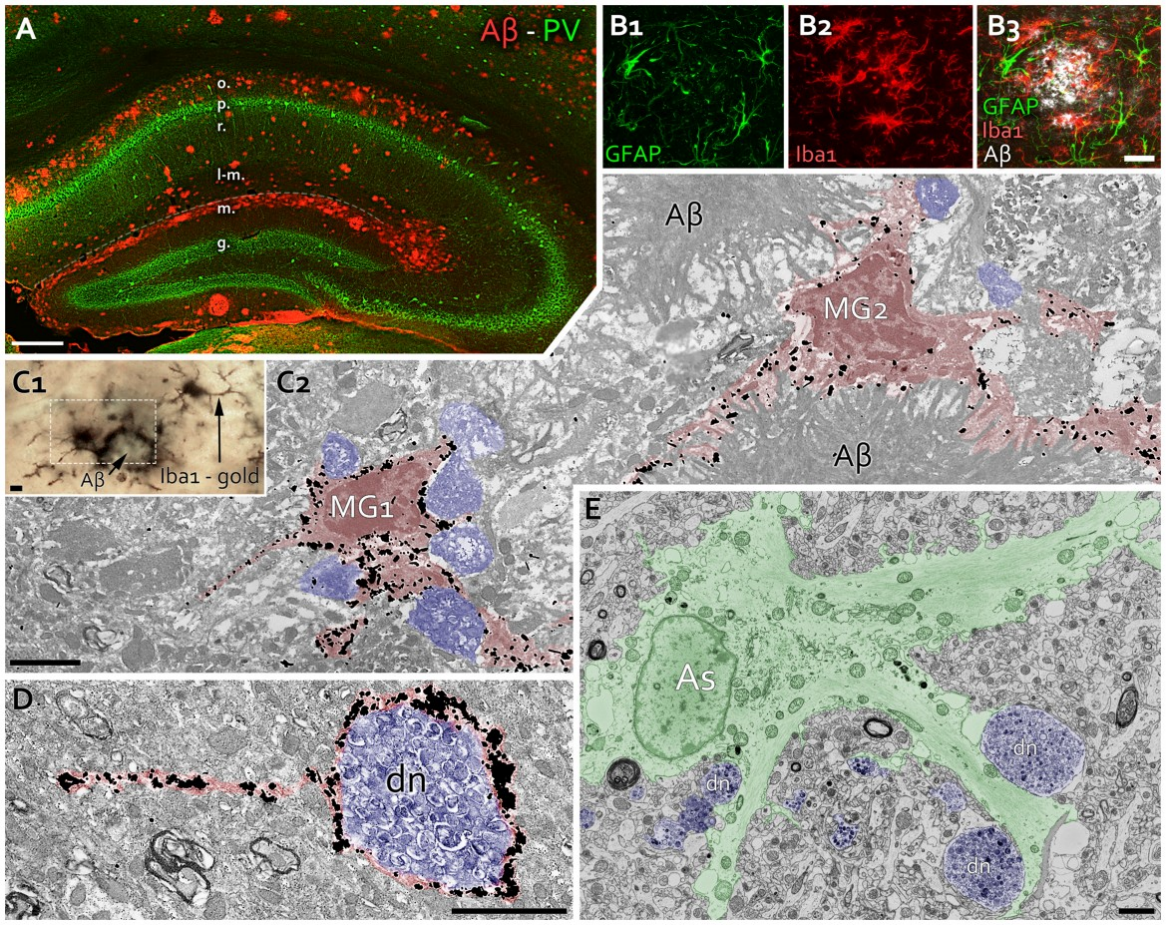
Rings of microglia and astroglia surround plaques and dystrophic neurites in APP^NL-F^ mice. **A:** Fluorescent image shows a hippocampal section of a 21-month-old APP^NL-F^ mouse. Aβ depositions (red) are abundant in strati oriens, lacunosum-moleculare and moleculare. PV-positive INs (green) seem unaffected. (o.: str. oriens, p.: str. pyramidale, r.: str. radiatum, l-m.: str. lacunosum-moleculare, m.: str. moleculare of dentate gyrus, g.: str. granulosum of dentate gyrus). Scale bar: 250 μm. **B**: Fluorescent images show GFAP-positive astrocytes (B1, green) and Iba1-positive microglia (B2, red) around an Aβ plaque (B3, white) in str. lacunosum-moleculare of a 20-month-old APP^NL-F^ mouse. Scale bar: 20 μm. **C:** Correlated light (C1) and electron microscopic images (C2 is from area with dashed line in C1) show Iba1 immunogold labeled microglia cells (MG1, MG2, red), which cover Aβ depositions and adjacent dystrophic neurites (blue). Scale bars: 2 μm. **D:** An Iba1 immunogold labeled microglial process (red) covers an autophagic vesicle-containing dystrophic neurite (dn, blue), separating it from the adjacent neuropil. Scale bar: 2 μm. **E:** Astroglial processes in an APP^NL-F^ mouse (As, green) separate dystrophic neurites (dn, blue) filled with electrondense autophagic materials, from the healthy-looking tissue. Scale bar: 2 μm.

We investigated whether a mutated humanized mouse APP gene, driven by the endogenous mouse promoter, is sufficient to induce typical amyloidosis and DN formation in mice. We used homozygous APP^NL-F^ mice and their WT littermates in all investigations. Using immunofluorescent staining, no Aβ accumulation was detectable in 7-month-old APP^NL-F^ mice. First plaques appeared at 9 months of age in several neocortical areas, then at around 12 months of age in the hippocampus. DNs were missing in 9-month-old APP^NL-F^ mice and were very rare in 12-month-old APP^NL-F^ mice. 18-21-month-old APP^NL-F^ mice showed amyloid plaque staining in all neocortical and hippocampal areas (Fig 1A), as described before [64]) and DNs were abundant around amyloid plaques.

Accumulation of Aβ is known to recruit both brain-resident microglia and astrocytes [76] and we also found ionized calcium-binding adaptor protein (Iba1)-positive activated microglial processes around amyloid plaques in str. lacunosum-moleculare of CA1 in 24-month-old APP^NL-F^ mice. Iba1-positive processes surrounded the edges of the plaques. DNs were also abundant around these microglia-Aβ complexes. Glial fibrillary acidic protein (GFAP) immunostaining also revealed the hypertrophy of astrocytes in the vicinity of amyloid plaques and DNs (Fig 1, S1 Fig).

**Fig 2:**
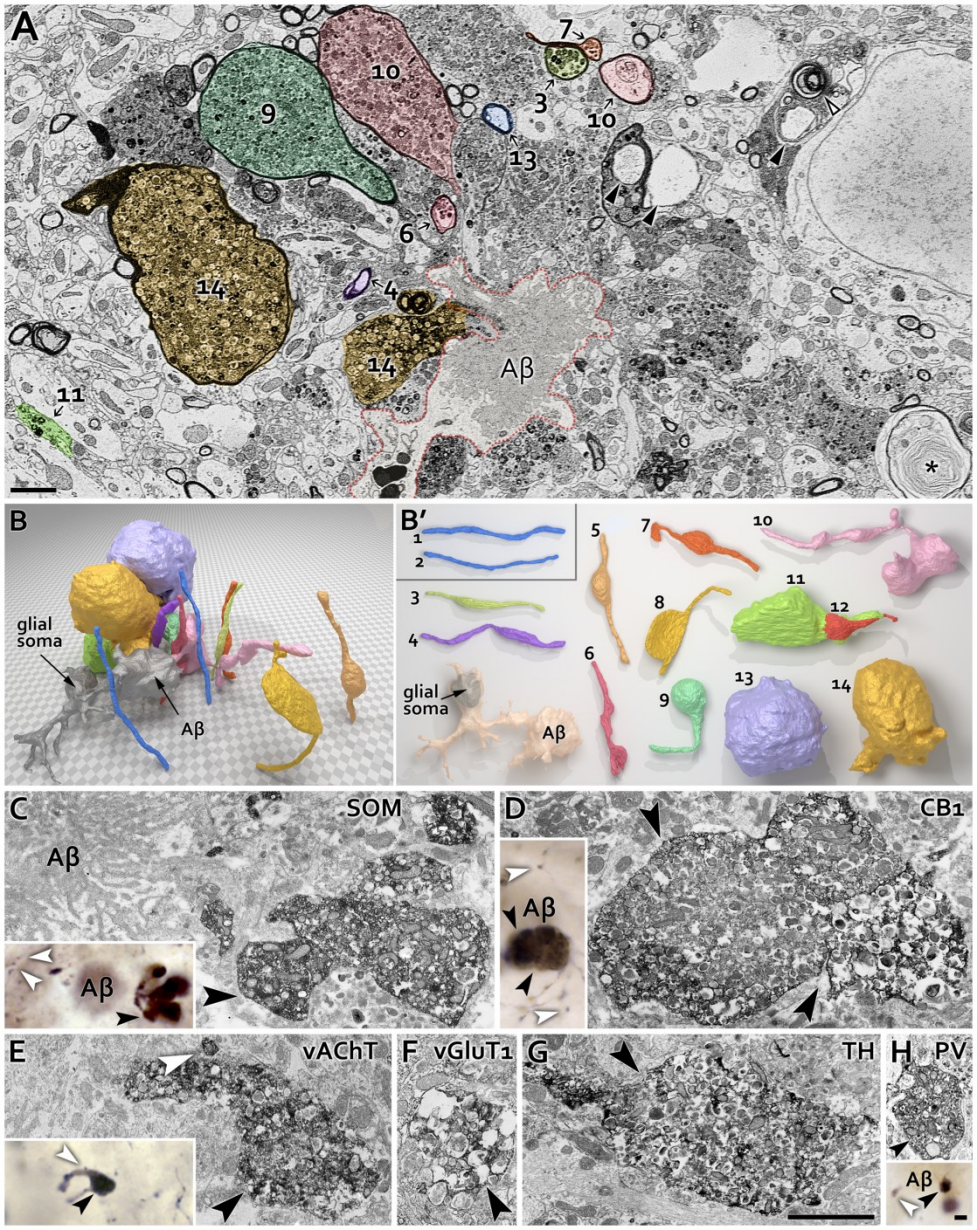
Diversely shaped dystrophic neurites and selective resilience of PV IN fibers in APP^NL-F^ mice. **A:** Representative electron microscopic image shows an Aβ plaque at the border of strati radiatum and lacunosum-moleculare from CA1 of 24-month-old APP^NL-F^ mouse. Several types of dystrophic neurites (DN) and Aβ-phagocyting microglia seem to be attracted to each other around the plaque center. Adjacent to DNs, there are different abnormal profiles e.g.: multilamellar bodies (*), vacuoles (black arrowheads) or split myelin sheaths (empty arrowhead). Amyloid deposits were detected in the extracellular space, and were also visible in microglia (dotted line). Scale bar: 2 μm. **B:** Large volume 3D reconstruction of a group of differentially affected axons (that regardless of their myelination were strongly dystrophic) and Aβ containing microglia (indicated on panel A). **B’:** Different stages of axonal degeneration (3–14, also on panel A) were visible around the plaque center (A, B). Some of them seemed healthy (1, 2), while other myelinated axons at least partially lost their myelin sheaths, mainly on the side that is facing to the amyloid-accumulation (10, 14). Also see S1 Video. **C-H:** Comparison of the sizes of DNs and healthy neurites of different types of neurons (DAB immunolabeling, dark precipitation) in 18-month-old APP^NL-F^ mice. Correlated light and electron micrographs show dystrophic terminals (black arrowheads) positive for somatostatin (SOM, C), cannabinoid type 1 receptor (CB_1_, D), vesicular acetylcholine transporter (vAChT, E), vesicular glutamate transporter 1 (vGluT1, F), tyrosine hydroxylase (TH, G) or parvalbumin (PV, H). White arrowheads indicate typical healthy-looking boutons. Light and electron micrographs that are on the same scale (respectively) demonstrate that most DNs are hundreds of times larger than healthy counterparts, however, the enlargement of PV-positive DNs were much less pronounced, while they were detected only very rarely. All scale bars: 2 μm.

**Fig 3:**
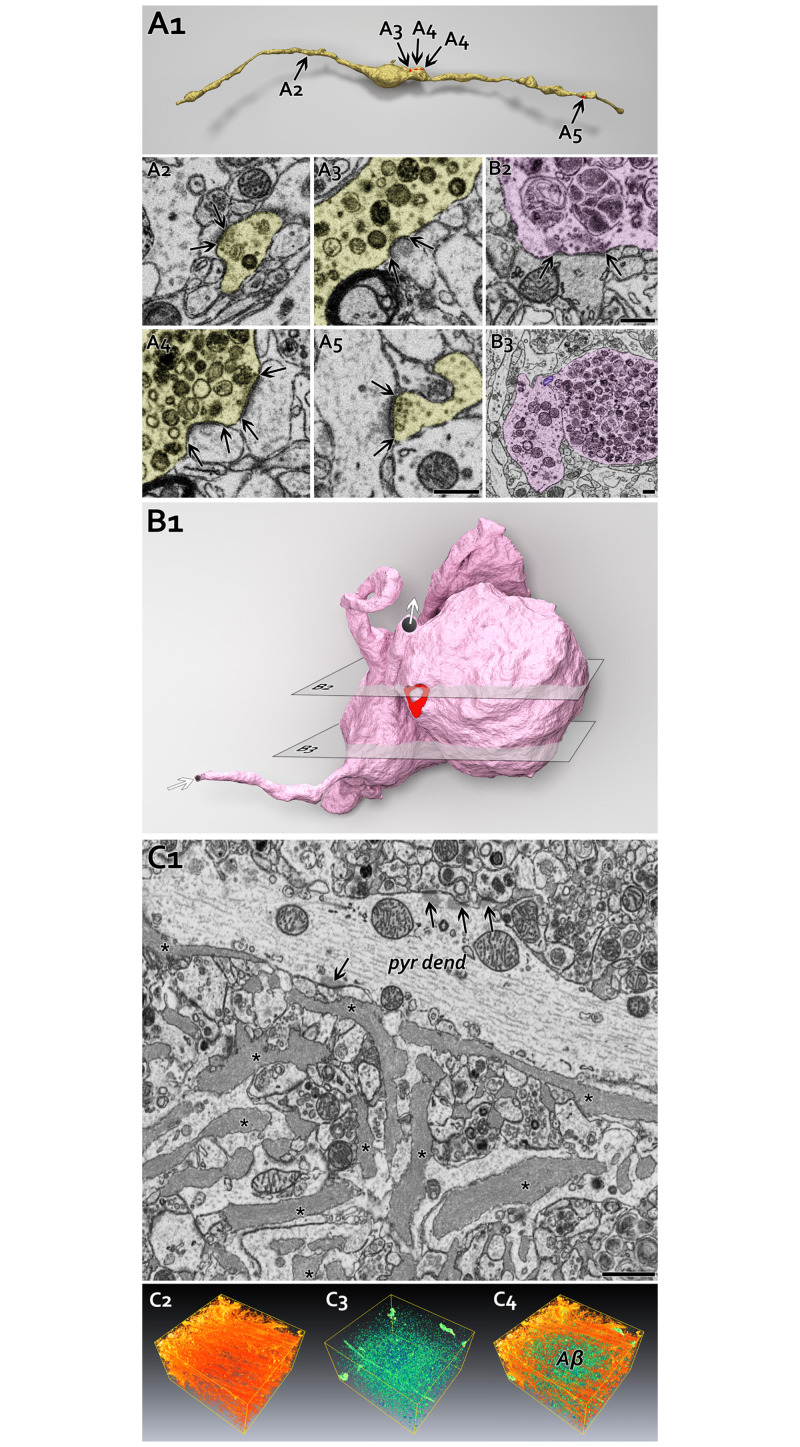
Subcellular structural alterations in dystrophic axons in APP^NL-F^ mice. **A:** 3D reconstruction of a mildly dystrophic axon (A1, yellow: axon, red: synapses). Black arrows indicate synapses, some of which are shown enlarged as well (A2-A5). Intracellular compartment accumulation (electron-dense dark material) around A3 and A4 synapses is clearly visible (on the 3D model: bulging middle part). Distant synapses (A2, A5) from the bulging part display seemingly normal morphology. (S2 Fig shows the surrounding tissue.) Scale bar: 500 nm. **B:** A balloon-like dystrophic axon (B1, pink: axon, red: synapses, white arrows indicate the axonal ends of this segment). B2, B3 show two representative section planes. Dystrophic axon generated three, almost detached balloons: one filled with normal looking but smaller mitochondria (main part, B3 left), the two others are filled with electron-dense degrading mitochondria and other cellular organelles (B3 right). Bulging dystrophic balloon has a perforated-like synapse that escaped microglial elimination (B2). Mitochondria seem to be transferred between balloons (B3, purple mitochondria). Scale bars: 500 nm. **C:** Filamentous Aβ accumulation (asterisks) around synapses in the stratum radiatum in hippocampus CA1 (C1). Arrows indicate synapses on pyramidal cell dendrite (pyr dend). C2-C3 shows 3D volume rendering from stratum radiatum. Pyramidal cell dendrites are orange-red colored (C2), whereas electron-dense dystrophic particles (auto-phagosomes, lysosomes, small, degrading mitochondria) colored teal (C3). Scale bar 1 μm. (Also see S2 Fig).

Using transmission and block-face scanning electron microscopy (BF-SEM), we investigated the ultrastructural signs of neuroinflammation and the presence of DNs in the neuropil of APP^NL-F^ mice at different ages. Using three-dimensional reconstructions of DNs, we investigated the morphology of dense-core plaques [27] at the border of str. radiatum and str. lacunosum-moleculare in CA1 of 24-month-old APP^NL-F^ mouse (Figs 2 and 3, S1 and S2 Figs). Amyloid deposits were located in the extracellular space, but it was visible also inside the microglia (Fig 2A) and different stages of degenerations were visible in neighboring axons. Some of them looked healthy and myelinated, whereas others lost their myelination partially (Fig 2). However, some other type of neuronal malformations were also present in 24-month-old WT littermates as well (S3 Fig). For instance, we found vacuoles in dendrites, myelin invagination into dendrites, redundant and disrupted myelin sheaths or empty myelinated profiles. However, only APP^NL-F^ mice had irregular mitochondrial and lysosomal accumulation, axonal swelling, neurites with clustered mitochondria and amyloid plaques with DNs [77].

### PV fibers are mostly spared form degeneration in APP^NL-F^ mice

In APP^NL-F^ mice, we frequently observed cholinergic (vAChT-positive), glutamatergic (vGluT1-positive) and dopaminergic [tyrosine hydroxylase (TH)-positive] DNs (Fig 2). However, we investigated hippocampal INs more closely, because previous human studies detected SOM within plaques and DNs [78,79], whereas cannabinoid receptor 1 (CB_1_R)-positive (PV-negative) INs were also shown to be sensitive to the cytotoxic effect of amyloid [80]. We found that 89% of the plaques (n = 45, 2 mice) had SOM-positive DNs, 93% of the plaques (n = 75, 2 mice) had CB_1_R-positive DNs, whereas only 4% of plaques (n = 93, 2 mice) had PV-positive DNs. In addition, those few PV-positive DNs showed much less bulging than any other types of DNs (Fig 2C–2H), suggesting that contrary to findings in other models, PV IN fibers seem to be more resilient to the effects of a mutant Aβ driven by an endogenous mouse promoter.

### Density of PV and SOM INs do not change significantly in APP^NL-F^ mice

**Fig 4:**
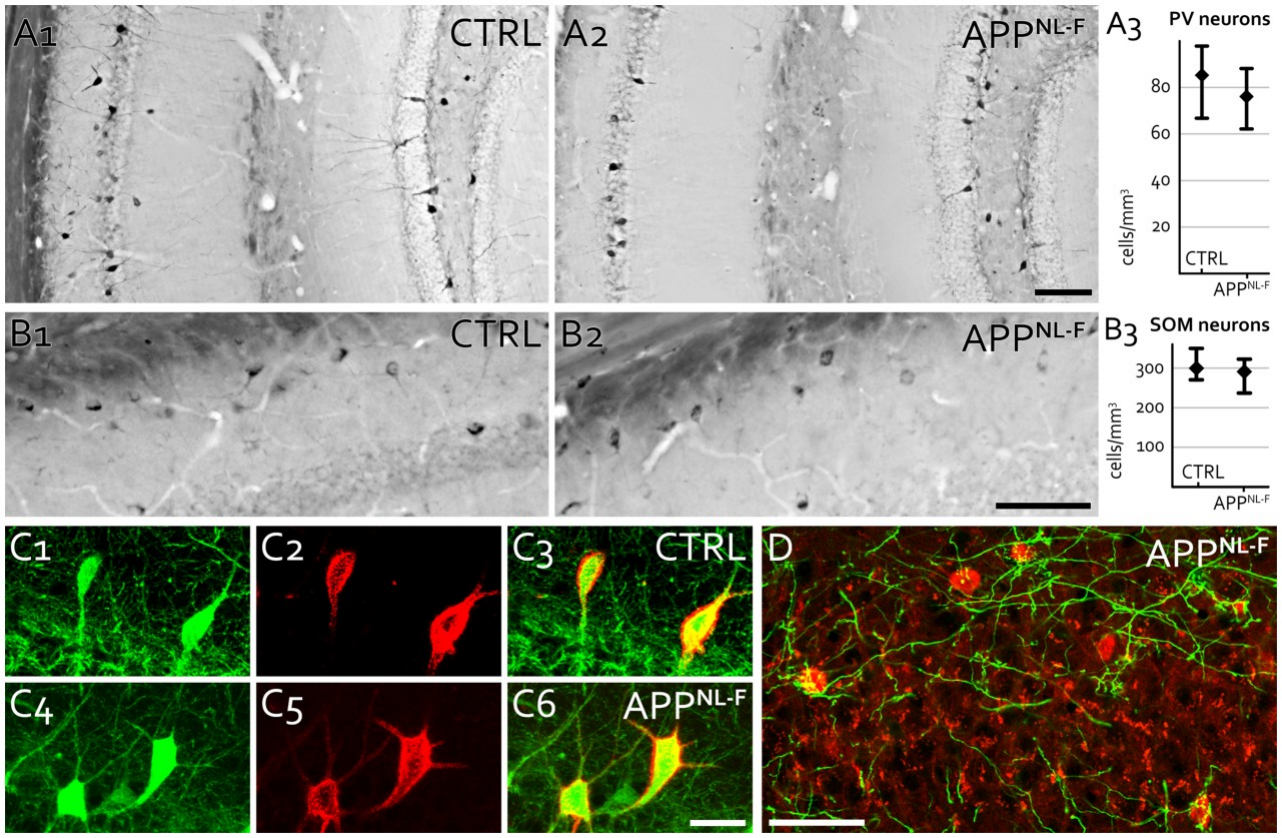
Investigation of local hippocampal interneurons in APP^NL-F^ mice. **A:** Hippocampal PV INs of 18-month-old WT (CTRL, A1) and APP^NL-F^ mice (A2). PV INs were labeled and counted using DAB immunolabeling in CA1-3 and dentate gyrus. No significant difference was found between genotypes (A3). Scale bar: 100 μm. **B:** Hippocampal SOM INs of 18-month-old control (CTRL, B1) and APP^NL-F^ mice (B2). SOM INs were labeled and counted using DAB immunolabeling in CA1 stratum oriens. No significant difference was found between genotypes (B3). Scale bar: 50 μm. **C:** PV INs (C1, C4, parvalbumin staining, green) are covered by perineuronal nets (C2-3, C5-6, PNN staining, red) both in 21-month-old WT (CTRL, C1-3) and APP^NL-F^ mice (C4-6). Scale bar: 25 μm. **D:** Virally traced septo-hippocampal PV positive input fibers (green) seem healthy and target local hippocampal PV INs (red) abundantly in 18-month-old APP^NL-F^ mice. Scale bar: 50 μm.

SOM-positive dendrite-targeting INs and PV-positive INs that mostly target pyramidal cells perisomatically have critical role in controlling hippocampal memory-formation and were considered to be vulnerable in several conventional models of amyloidosis [56,81]. Using stereology-based methods, we found that the density of PV neurons did not change significantly in the hippocampus (including CA1-3 and dentate gyrus) of 18–20 months old APP^NL-F^ mice compared to WT littermates (Fig 4A, Mann-Whitney test: p = 0.6625, 85 (67–98) cells/mm^3^ in 3 WT mice vs. 76 (62–88) cells/mm^3^ in 3 APP-NL-F mice, median (lower-upper quartile).

In addition, no significant change was detected in the density of SOM INs of APP^NL-F^ mice compared to their WT littermates in the CA1 stratum oriens of 24-month-old APP^NL-F^ mice (Fig 4B, Mann-Whitney test: p = 0.6625, 300 (270–350) cells/mm^3^ in 3 WT mice vs. 291 (237–322) cells/mm^3^ in 3 APP-NL-F mice, median (lower-upper quartile).

We also tested whether axonal fibers of septo-hippocampal PV-positive projecting neurons show any sign of degradation in the hippocampus, but we found no change in their target specificity or abundance (Fig 4D).

### PV IN-specific perineuronal nets do not change in APP^NL-F^ mice

Although malformation of the protective perineuronal net (PNN) coverage of mature hippocampal PV neurons has been found in some APP overexpressing models of AD [82–84], it seems unaffected in human patients [85]. Using fluorescent immunohistochemistry, we qualitatively investigated PNN formation in the hippocampi of 9-, 12-, 21-month-old APP^NL-F^ mice versus their WT littermates, but found no evidence of PNN deterioration (Fig 4C).

### GABAergic synaptic clefts do not show significant differences in APP^NL-F^ mice

**Fig 5:**
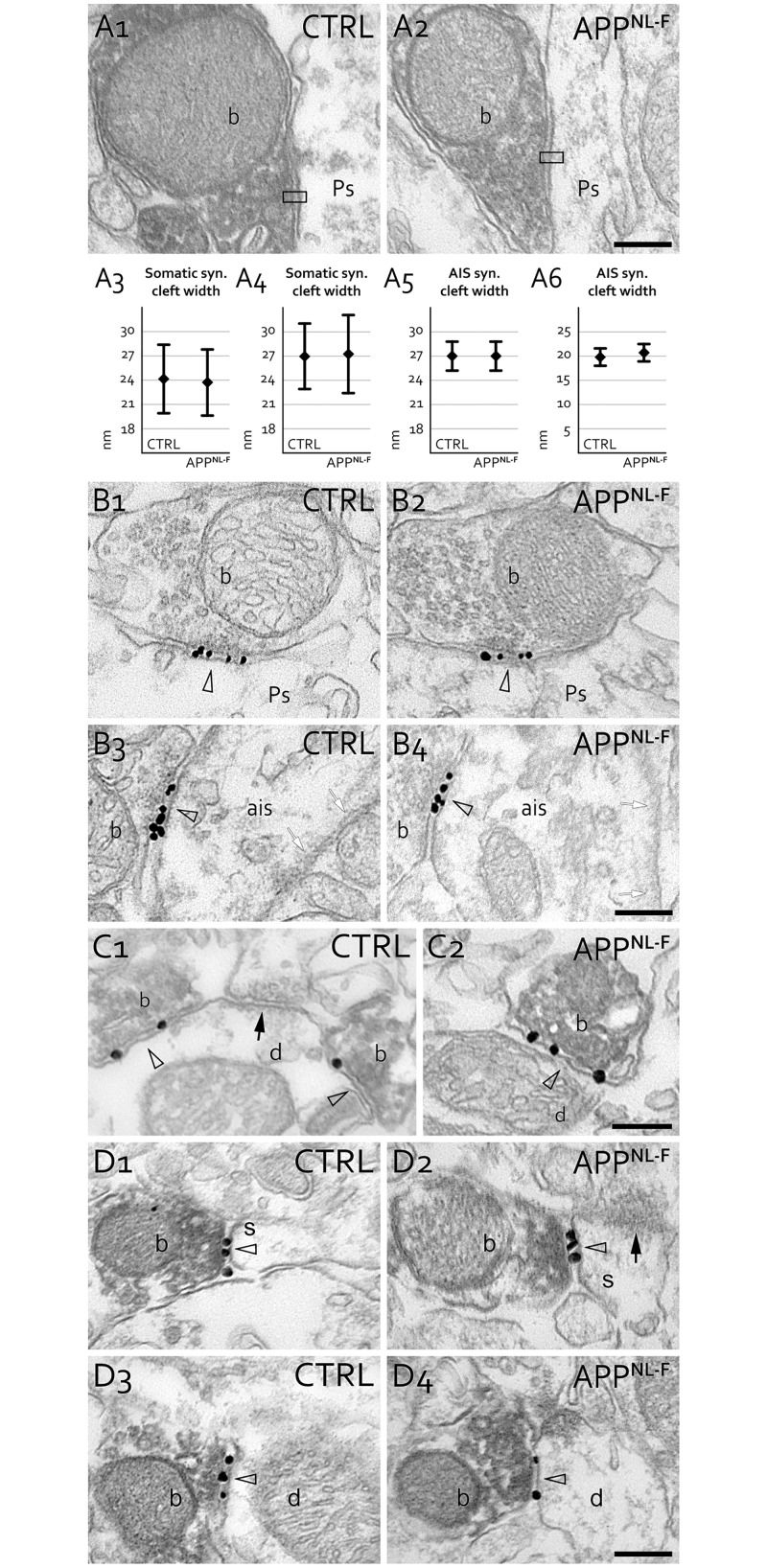
Synapses and GABA_A_ receptors of hippocampal inhibitory synapses in APP^NL-F^ mice. **A:** Synaptic cleft width (profiles tested in black frame) of PV-positive (DAB dark precipitate) somatic or AIS terminals (b: bouton) on CA1 pyramidal cell somata (A1-2, Ps: pyramidal cell some) or AIS in 18-month-old APP^NL-F^ mice were not significantly different on somata (in two pairs of mice, A3-4) or on AISs (in two pairs of mice, A5-6) compared to WT mice (CTRL). Scale bar: 200 nm. **B:** Immunogold-labeled GABA_A_ γ2 receptor subunit localization in the synapses (arrowheads) established by somatic terminals (B1, B2) and terminals innervating axon initial segments (ais, B3, B4) in 17- and 21-month-old WT (CTRL) and APP^NL-F^ mice, respectively. No changes were found in their GABA_A_ γ2 receptor content. Small white arrows indicate AIS specific membrane undercoating. Scale bar: 200 nm. **C:** Synaptic GABA_A_ receptor labeling of SOM-positive terminals is similar in WT (CTRL, C1) and APP^NL-F^ mice (C2). Electron micrographs show immunogold-labeled GABA_A_ γ2 receptor subunits in synapses (arrowheads) established by SOM positive terminal boutons (b, labeled with DAB) on postsynaptic dendrites (d) in CA1 stratum lacunosum-moleculare of 17-month-old mice. Black arrow shows an unlabeled synaptic contact of a SOM-negative bouton (C1). Scale bar: 200 nm. **D:** The GABA_A_ receptor labeling of cholinergic synapses in the hippocampus is similar in WT (CTRL, D1, D3) and APP^NL-F^ (D2, D4) littermate mice. Electron micrographs show immunogold-labeled GABA_A_ γ2 receptor subunits in synapses (open arrowheads) established by vesicular acetylcholine transporter (vAChT)-positive cholinergic boutons (b, labeled with DAB) innervating spines (s, D1-D2) or dendrites (d, D3-D4) in CA1 of WT (CTRL) and APP^NL-F^ mice. Black arrow in D2 shows the asymmetric excitatory input of the same spine. Scale bar: 200 nm.

Inhibitory synapses and GABA_A_ receptors were reported to be affected in AD (Limon et al., 2012; Ulrich, 2015) and changes in synaptic plasticity can influence synaptic adhesion and scaffolding and can therefore influence cleft width. Therefore, we compared these features between WT and APP^NL-F^ animals. We measured the width of the inhibitory synaptic cleft of somatic terminals on pyramidal cells in CA1 and found no difference between pairs of WT and APP^NL-F^ mice [Fig 5A, first pair of mice, t-test: p = 0.64, 24.1 ± 4.2 nm (39 WT synapses) vs. 23.7 ± 4.1 nm (42 APP^NL-F^ synapses); second pair of mice, t-test: p = 0.78, 27.0 ± 4.1 nm (48 WT synapses) vs. 27.3 ± 4.8 nm (36 APP^NL-F^ synapses), mean ± SD]. We also measured the width of cleft of AIS synapses on pyramidal cells in CA1 and found no difference between pairs of WT and APP^NL-F^ mice [Fig 5, first pair of mice, Mann-Whitney test: p = 0.77, 27 (25.2–28.8) nm in 17 WT synapses vs. 27 (25.2–28.8) in 21 APP^NL-F^ synapses, median (lower-upper quartile); second pair of mice, Mann-Whitney test: p = 0.64, 19.8 (18–21.6) nm in 8 WT synapses vs. 20.7 (18.9–22.5) nm in 8 APP^NL-F^ synapses].

### GABA_A_ receptor content does not show significant differences in APP^NL-F^ mice

We measured the ratio of GABA_A_ receptor γ2 subunit positive synapses of PV IN terminals on pyramidal cells in CA1 in 2 WT and 2 APP^NL-F^, 17- and 21-month-old mice. We found that practically all synapses established by PV positive somatic terminals (WT: 68/69, APP^NL-F^: 68/68), by PV positive axo-axonic INs terminals [WT: 45/45 on 9 axon initial segments (AIS), APP^NL-F^: 61/62 on 13 AISs] and by SOM IN terminals (WT: 19/19, APP^NL-F^: 29/29) were GABA_A_ receptor γ2 subunit positive (Fig 5B and 5C).

Because we previously demonstrated that hippocampal cholinergic terminals establish GABAergic synapses [86], and because cholinergic system is known to be especially vulnerable in AD, we investigated their GABA_A_ receptor γ2 subunit positivity as well. Again, we found no difference between WT and APP^NL-F^ mice (Fig 5D, WT: 33/41, 81%; APP^NL-F^: 36/44, 82%).

### Inhibitory synaptic area is affected in a cell type specific manner in APP^NL-F^ mice

Larger synapses contain more receptors and are more effective [87]. Using BF-SEM, we fully reconstructed inhibitory synapses of SOM-positive distal dendrite-targeting INs, PV-positive (CB_1_R-negative) and PV-negative (CB_1_R-positive) soma-targeting basket cells and PV-positive AIS-targeting axo-axonic INs that establish synapses on CA1 pyramidal cells.

**Fig 6:**
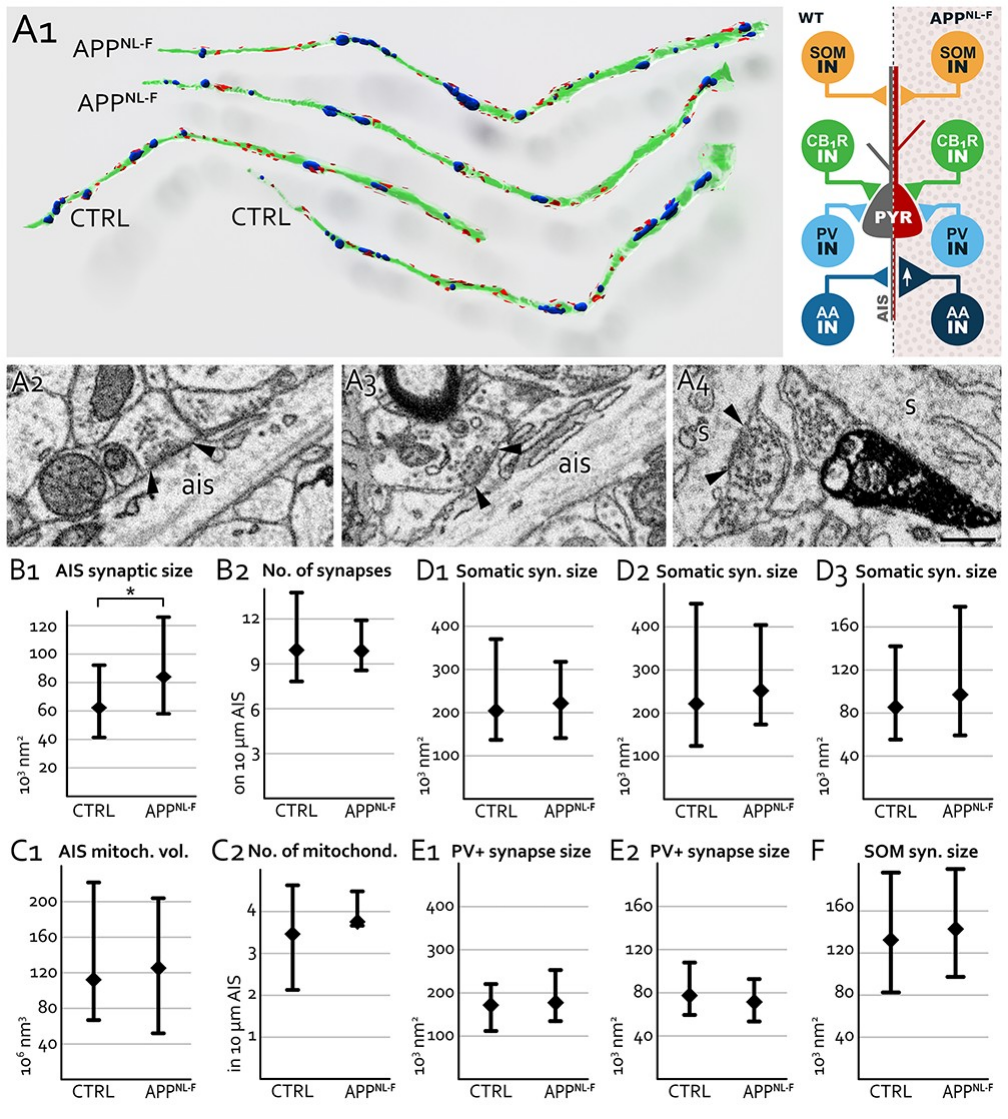
3D morphology of hippocampal inhibitory synapses in 18-month-old APP^NL-F^ mice. **A:** BF-SEM 3D reconstruction of CA1 pyramidal cell axon initial segments (AISs) from APPNL-F and WT (CTRL) animals. Synapses (red) on the axonal membrane (green) and mitochondria (blue) were also reconstructed and counted. On the right hand side, schematic representation of the results show that while synaptic area of SOM-positive INs (SOM IN), CB1R-positive basket cells (CB1R IN), PV-positive basket cells (pale blue PV IN) do not change, synaptic area of PV-positive axo-axonic cells (AA IN) enlarged significantly by about 35% (A1). Representative BF-SEM images show synapses (black arrows at synaptic edges) formed by axo-axonic terminals on pyramidal cell axon initial segments (ais) of APP^NL-F^ (A2) and WT (A3) mice. In addition, CB_1_R-negative somatic terminals on pyramidal cells (s) in CA1 (dark precipitation indicates CB1R-staining, A4). **B:** Significant increase was found in the size of pyramidal cell AIS synapses in three pairs (pooled data) of APP^NL-F^ and WT (CTRL, B1) mice, while the linear density of their synapses on AISs remained unchanged (B2). **C:** No changes in the size (C1) or density (C2) of pyramidal cell mitochondria in the AISs as detected in three pairs (pooled data) of WT (CTRL) and APP^NL-F^ mice. **D:** Sizes of synapses on pyramidal cells somata did not change in three pairs of WT and APP^NL-F^ mice (D1-3). **E:** Sizes of synapses established by PV-positive (CB_1_R-negative) INs on pyramidal cells somata were not different in two pairs of 18-month-old WT and APP^NL-F^ mice (E1, E2). **F:** Sizes of synapses established by SOM-positive INs on pyramidal cells dendrites were not different in two pairs (pooled data) of 18-month-old WT and APP^NL-F^ mice.

First, we compared all types of somatic basket cell synapses together in 18-month-old WT and APP^NL-F^ mice (Fig 6). We found no differences in the area of their synapse (because of differences in shrinkage, mice were processed and compared pairwise, see Table 3 for details). Then, using CB_1_R immunogold labelling, we differentiated the two types of basket cell terminals and specifically determined the sizes of synapses established by CB_1_R-negative (PV-positive) terminals [88]. We found that synapses of PV-positive (CB_1_R-negative) basket cells did not change in APP^NL-F^ mice (see Table 3 for details). Then we compared the sizes of synapses established by SOM IN on distal dendrites of pyramidal cells and again found no difference between WT and APP^NL-F^ littermate mice (Fig 6 and see Table 3 for details).

**Table 3:** Statistical data for BF-SEM experiments.

Ultrastructural parameters	WT	APP^NL-F^
Synaptic area of somatic synapses on CA1 pyramidal cells (nm^2^, mouse pair 1), p = 0.9542		
Number of synapses	100	100
Median	203898	221232
Lower quartile	136960	141059
Upper quartile	370260	317769
Synaptic area of somatic synapses on CA1 pyramidal cells (nm^2^, mouse pair 2), p = 0.3388		
Number of synapses	100	100
Median	221674	251685
Lower quartile	123681	173417
Upper quartile	453867	404389
Synaptic area of somatic synapses on CA1 pyramidal cells (nm^2^, mouse pair 3), p = 0.4349		
Number of synapses	173	177
Median	85496	96940
Lower quartile	55382	59209
Upper quartile	142013	178778
Synaptic area of CB_1_R-negative PV INs on CA1 pyramidal cell somata (nm^2^, mouse pair 4), p = 0.1860		
Number of synapses	90	100
Median	171460	177651
Lower quartile	112007	134771
Upper quartile	220626	253022
Synaptic area of CB_1_R-negative PV INs on CA1 pyramidal cell somata (nm^2^, mouse pair 5), p = 0.0993		
Number of synapses	100	100
Median	77320	71605
Lower quartile	59435	53437
Upper quartile	107981	92711
Synaptic area of SOM INs on pyramidal cell dendrites (nm^2^) collected from two pairs of mice, p = 0.0706		
Number of synapses	194	196
Median	132486	142723
Lower quartile	82546	97368
Upper quartile	196627	200001
**Synaptic area on pyramidal cell AISs (nm**^**2**^**) collected from three pairs of mice, p = 5,7038239708077E-10**		
Number of synapses	387	380
Number of AIS	9	9
Median	62096	83983
Lower quartile	41403	58021
Upper quartile	92186	125968
Volume of mitochondria of pyramidal cell AIS (nm^3^ x 10^6^) collected from three pairs of mice, p = 0.7535		
Number of mitochondria	101	124
Number of AIS	8	8
Median	112	125
Lower quartile	67	52
Upper quartile	222	204
Linear density of synapses on AIS (number/10μm) collected from three pairs of mice, p = 0.9755		
Number of synapses	460	515
Number of AIS	12	11
Median	9.91	9.85
Lower quartile	7.83	8.56
Upper quartile	13.77	11.91
Density of mitochondria of pyramidal cell AIS (number/10μm) collected from three pairs of mice, p = 0.4705		
Number of mitochondria	100	138
Number of AIS	8	9
Median	3.46	3.75
Lower quartile	2.13	3.66
Upper quartile	4.63	4.48

Aβ causes increased glutamate release probability [28,70] and subsequently higher pyramidal cell activity, which can most effectively be compensated by the inhibition of AISs that have a crucial role in the generation of action potentials in pyramidal cells. Therefore, using BF-SEM, we specifically investigated several morphological features of AISs in three pairs of 18-month-old WT and APP^NL-F^ mice and we fully reconstructed several AISs from each of these six mice (Table 3). We found changes neither in the average density of synapses on AISs, nor in the density and volume of mitochondria inside AISs (see Table 3 for details). However, we found that synapses on AISs were significantly larger in APP^NL-F^ mice by about 35% (Mann-Whitney test: p<0,001, Fig 6 and see Table 3 for details).

### Natural anxiety is suppressed in APP^NL-F^ mice

**Fig 7:**
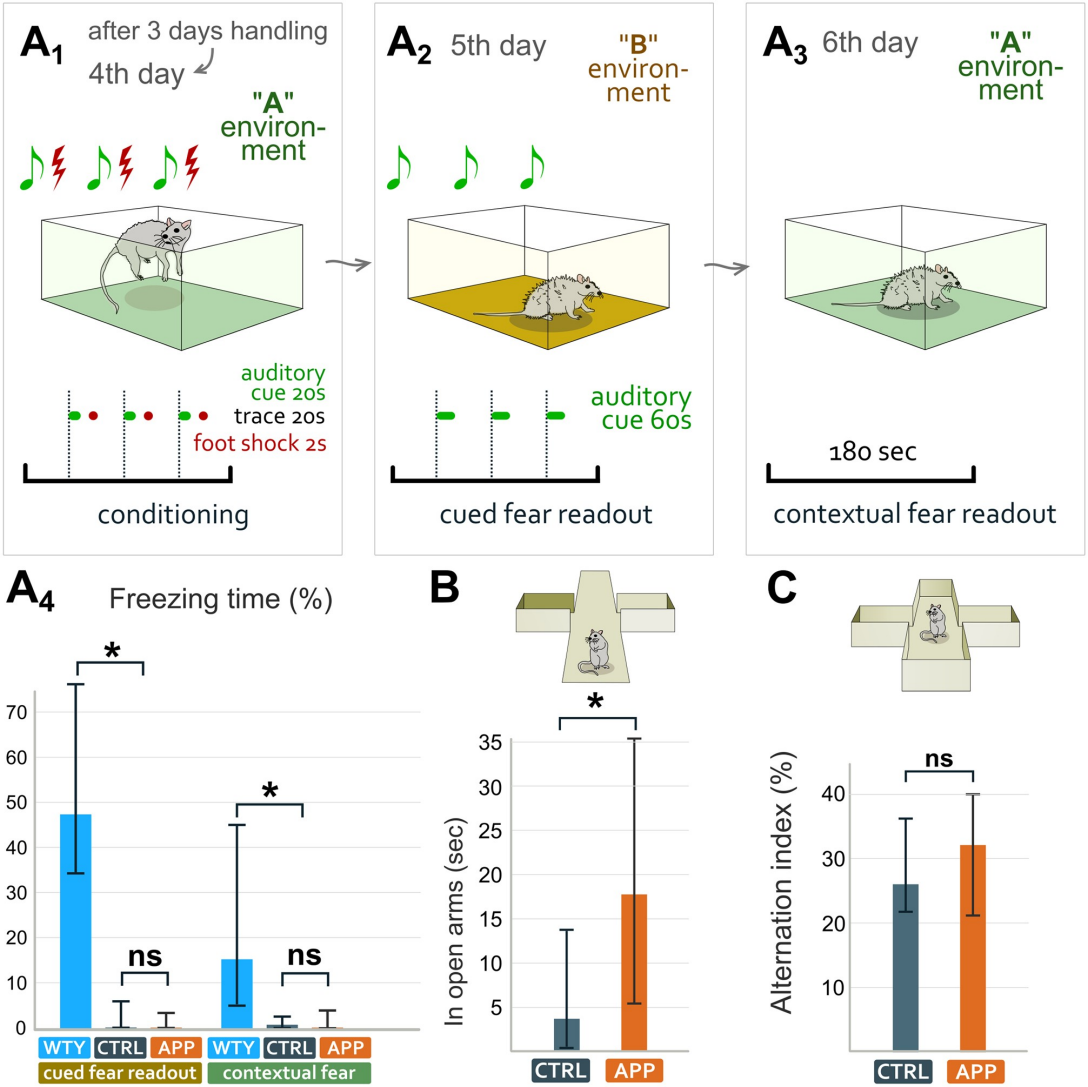
Behavioral examination of APP^NL-F^ mice. **A:** Contextual and cued fear memory of APP^NL-F^ mice was tested in the trace fear conditioning paradigm, where the neutral tone and the aversive shocks were separated by a 20 sec trace interval (A1), that was followed by testing cued fear (A2) and contextual fear memory (A3) on the subsequent 2 days. 15-19-month-old APP^NL-F^ and littermate WT (CTRL) mice both showed impaired fear learning with very low amount of freezing behavior compared to 3-month-old young wild type (WTY) mice (A4). **B:** 15-19-month-old APP^NL-F^ mice spent significantly more time in open arms than WT (CTRL) littermates during the elevated plus maze paradigm. **C:** In the spontaneous alternation paradigm 15-19-month-old APP^NL-F^ and littermate WT (CTRL) mice performed similar amount of perfect alternations of ABCD type.

Besides contextual memory formation, hippocampus plays a role in anxiety as well [89] and indeed, amyloidosis has already been suggested to influence anxiety [66]. Therefore, 15-19-month-old WT and APP^NL-F^ mice were tested in elevated plus maze for 5 min (Fig 7). We found that APP^NL-F^ mice spent significantly more time in the open arm [Mann-Whitney-test: p = 0.0313; WT: 3.66 (0.4–13.74) sec in 16 mice; APP^NL-F^: 17.76 (5.4–35.4) sec in 19 mice, median (lower-upper quartile)] and consequently they also travelled a longer distance while exploring the context [t-test: p = 0.0297; WT: 841 ± 267 cm; APP^NL-F^: 1082 ± 345 cm, (mean ± SD)]. Lower level of anxiety in APP^NL-F^ mice may suggest a deterioration in the effective recognition of a potentially dangerous context or abnormalities in visual function in APP^NL-F^ mice [90,91].

### No alteration of spatial working memory was detected in APP^NL-F^ mice

The hippocampus also plays a role in the formation of spatial working memory that we tested in 15-19-month-old WT and APP^NL-F^ mice using spontaneous alternation test in a four arm plus maze (Fig 7). We found no significant difference between the two groups that performed similar proportion of perfect alternations [ABCD type, Mann-Whitney-test: p = 0.9077, WT: 26% (21.8–36.2%) in 16 mice; APP^NL-F^: 32% (21.2–40%) in 19 mice, median (lower-upper quartile)], similar proportion of mistakes of the AA type [p = 0.3625; WT: 2.67% (1.7–8.4%); APP^NL-F^: 3.13% (0–5.8%)], the ABA type (p = 0.0469; WT: 18.8% (13.4–27.1%); APP^NL-F^: 26.4% (22.6–31.4%)], and the ABCA type [p = 0.1909; WT: 21.6% (19.5–30.2%); APP^NL-F^: 20.7% (16.7–23.8%)]. There was no difference in the total distance travelled (Mann-Whitney Test: p = 0.2143; WT: 27.8 (25.1–30.1) m; APP^NL-F^: 34.0 (26.0–36.9) m, median (lower-upper quartile). Similar to our results, Saito et al did not find memory impairment in Morris water maze test of APP^NL-F^ mice either [64].

### No alteration of fear memory was detected in APP^NL-F^ mice

Using trace fear conditioning paradigm, we tested a more complex hippocampal function in 15-19-month-old WT and APP^NL-F^ mice (Fig 7). We presented mice with a neutral conditioned stimulus (20 s auditory cue) and the aversive unconditioned stimulus (2 s footshock), separated by a 20 s trace interval. On the next day, we presented mice with the auditory cue alone to test their cued fear level, and found no difference between the two groups [Mann-Whitney Test: p = 0.4562, WT: 0% (0–5.9%) in 16 mice; APP^NL-F^: 0% (0–3.3%) in 19 mice, median (lower-upper quartile)]. On the next day, we placed them back into the same context, where they previously received the footshocks to test their contextual fear level and again, we found no difference [p = 1,0000 WT: 0.7% (0–2.6%); APP^NL-F^: 0% (0–3.9%)]. However, because memory performance declines with age even in WT mice, in parallel, we also tested young, 3-month-old mice (WTY) in the same cohort. These tests showed that even without genetic modification older mice perform significantly worse in both cued fear test [p = 0.0009, WTY (3 months old): 47.2% (34.3–76.3%) in 10 mice; WT (15-19-month-old): 0% (0–5.9%) in 16 mice] and in contextual fear test [p = 0.0106, WTY: 15.2% (5.0–44.9%); WT: 0.7% (0–2.6%)]. These results indicate that fear memory performance can decline significantly with age as demonstrated previously [92].

## Discussion

AD is diagnosed postmortem by the presence of amyloid plaques in the patient’s brain [93]. Although mutations of several proteins, including tau-protein, apolipoprotein E, presenilin have already been found in severe forms of dementias, for decades, amyloid plaques were considered one of the primary causes of AD [7–9]. Aβ was therefore the primary target for removal from the brain in animal experiments [10–13] and even in several clinical trials [10,17]. This theory inspired the creation of over a hundred mouse models, in which accumulation of Aβ was induced by the overexpression of mutated APP driven by a strong non-specific promoter. These approaches sped up investigations of possible effects of Aβ, because they could be carried out in a few months after birth. However, these mouse models could express mutated APP in more cells, in artificially high quantities and could introduce model-specific artefacts. Here, we investigated the combined effect of the Swedish and Iberian mutations, which are common in patients with familial AD [57]. APP^NL-F^ mice express the Swedish and the Iberian mutations of the human APP driven by the endogenous natural mouse APP promoter and express Aβ42 and Aβ40 isoforms in a ratio that is typical in human AD. This makes APP^NL-F^ mice a more realistic model to study the effect of Aβ on the brain [64].

Cognitive decline and memory impairment is not detectable in the early years of human AD, but develops progressively [94,95]. We found that working memory of 15–19 months old APP^NL-F^ mice seem to be unaffected and also impairment of fear memory learning was similar in WT and APP^NL-F^ mice. Indeed, people without apparent cognitive dysfunction, can also have heavy Aβ plaque load [5,20–24]. However, a decline in judgmental ability of threats were also observed in AD patients [96] and consistent with this, we found that APP^NL-F^ mice showed unnaturally low anxiety in elevated plus maze test.

Contrary to findings in APP overexpressing model mice with APP driven by strong non-specific promoters and loss of hippocampal SOM- [51–53] and PV-positive INs [54–56], we found no significant decline in the density of these INs. Damage of the perineuronal nets around PV neurons were also reported [82–84], but we detected no apparent perineuronal net damage in APP^NL-F^ mice, similar to findings in humans [85]. While GABA_A_ receptors were known to be affected in AD [97,98] we found no effect on synaptic cleft width of inhibitory synapses or on GABA_A_ receptor content of somatic GABAergic synapses and cholinergic synapses. Comparison of different anatomical properties of other three types of APP-expressing and APP^NL-F^ model mice were summarized in S1 Table.

However, we found that amyloid plaque formation did have a local neurotoxic inflammatory-like effect because in the immediate vicinity of plaques we observed typical microglial and astroglial reactions and DN formations. In human, Aβ depositions cause glial activation, dystrophic axonal processes and aberrations in neurotransmitter systems [31,99,100] that leads to the slow development of AD [95]. Microglia seemed to be effective in preventing the spreading effect of Aβ around plaques in APP^NL-F^ mice because DN formation was confined to a relatively small volume around the plaque center. We found irregular mitochondria, lysosomal accumulation, axonal swelling, and clustered mitochondria in DNs, that are likely caused by axonal damage-related calcium-influx and subsequent mitochondrial dysfunction [101,102]. This probably hampered mitochondrial movement, function, and more importantly mitochondrial calcium buffering [103,104] that prevented axonal transport at least locally [5]. Several of these alterations have also been observed in humans with AD [105,106]. Most types of neurons produced DNs in APP^NL-F^ mice because we observed huge cholinergic, glutamatergic, dopaminergic, SOM- and CB_1_R-positive GABAergic dystrophic boutons as well. However, several age-related morphological changes were also detected in WT littermate mice, and cued and contextual fear memory of old mice were also severely affected by age, regardless of amyloidosis.

Because Aβ causes increased glutamate release probability [28,70] and a subsequently higher pyramidal cell activity, network hyperexcitability is also frequent in humans with Swedish or Iberian familial AD mutation [57]. Local inflammatory reaction-related cytokine-release [107] can facilitate epileptiform activity in AD patients in the early stages of the disease [58,108,109]. However, higher pyramidal cell activity can also induce homeostatic compensatory mechanisms in local INs [110,111] that can effectively inhibit pyramidal cell firing. We found that PV neurons would be ideal for this task, because they are more resilient to Aβ-related challenges, many of these fast spiking INs have very effective calcium buffering capacity (parvalbumin itself is an effective calcium-binding protein in PV neuron [112,113]) and they have mitochondrial density that is typically higher than in most other INs [114,115]. We found that healthy-looking PV terminals were abundant around plaques and PV-positive DNs were detected very rarely. PV IN density was unchanged and their synapses remained positive for GABA_A_ receptor subunits. We also found that expression of perineuronal nets around PV neurons were unaffected in 18-month-old APP^NL-F^ mice. Therefore, we also investigated synaptic strength of four different populations of hippocampal INs. Because synaptic strength and area correlates strongly [87], we estimated the synaptic areas of INs that inhibit pyramidal cell activity. We found that the size of synapses of dendrite targeting SOM INs, soma-targeting PV- and CB_1_R-positive basket cells did not change. However, the synapses on AISs established exclusively by PV-positive axo-axonic cells [116,117] were significantly larger in APP^NL-F^ mice by about 35%.

These results suggest that contrary to data from other AD models with overexpression of APP, a more natural development of pure amyloidosis spares PV-positive INs even in relatively old APP^NL-F^ mice. Furthermore, PV-positive axo-axonic cells may effectively counteract higher pyramidal cell activity in APP^NL-F^ mice. These results also suggest that mechanisms that help the survival of PV neurons could be exploited in future treatments because those mechanisms can support the survival of other cells as well and can help mitigating Aβ-related inflammatory effects on neurons.

## Supporting information

S1 Fig Localization of different types of glial cells around dystrophic neurites and amyloid-beta plaque. Representative correlated light (inset) and electron micrographs shows that an amyloid plaque is surrounded by microglia cells (MG, red) and GFAP-positive (DAB-labeled) astrocytes (green). Dystrophic neurites are located in the vicinity of the amyloid deposition bordered by astrocytes (As, green) which seem to separate damaged area from the environment. Scale bar: 4um.
(TIF)

S2 Fig Surrounding area of profiles reconstructed in Fig 2. **A:** Representative section shows the environment of a mildly (B, yellow) and a strongly (C, pink) dystrophic axons described in Fig 2. (As: astrocyte, green; MG: microglia, red). **B:** Dendrites (pyr dend) of pyramidal cells in the stratum radiatum from hippocampus CA1. Aβ filaments build up around dendrites as a thin layer. Dashed area is presented in Fig 3C. Scale bar: 500 nm.
(TIF)

S3 Fig Morphological alternations are not unique to APP^NL-F^ mice at 24 months age. In hippocampus, both WT and APP^NL-F^ mice had vacuoles-like profiles in dendrites (A), myelin sheaths bulging into dendrite (B), split myelin sheaths (C), empty-looking myelinated balloons (D), myelinated balloons with axon-like profiles (E), redundant myelination (F), disrupted axonal myelin (G) and gliosis (H). However, WT mice very rarely had mitochondria and lysosome accumulations (J), axonal swellings (K) and neurites with clustered mitochondrial core (L) that were typical in APP^NL-F^ mice. Finally, amyloid plaques with dystrophic neurites were present only in APP^NL-F^ mice (I).
(TIF)

S1 Video Reconstructed dystrophic neurites of Fig 2B. (MP4)

S1 Table. Com parisons of different anatomical properties of APP-expressing model mice. (DOCX)
