# Supplementary figures and images for: Amyloid β induces interneuron-specific changes in the hippocampus of APPNL-F mice
Source: PLoS One. 2020 May 29;15(5):e0233700. doi: 10.1371/journal.pone.0233700 (PMC7259556; doi:10.1371/journal.pone.0233700)

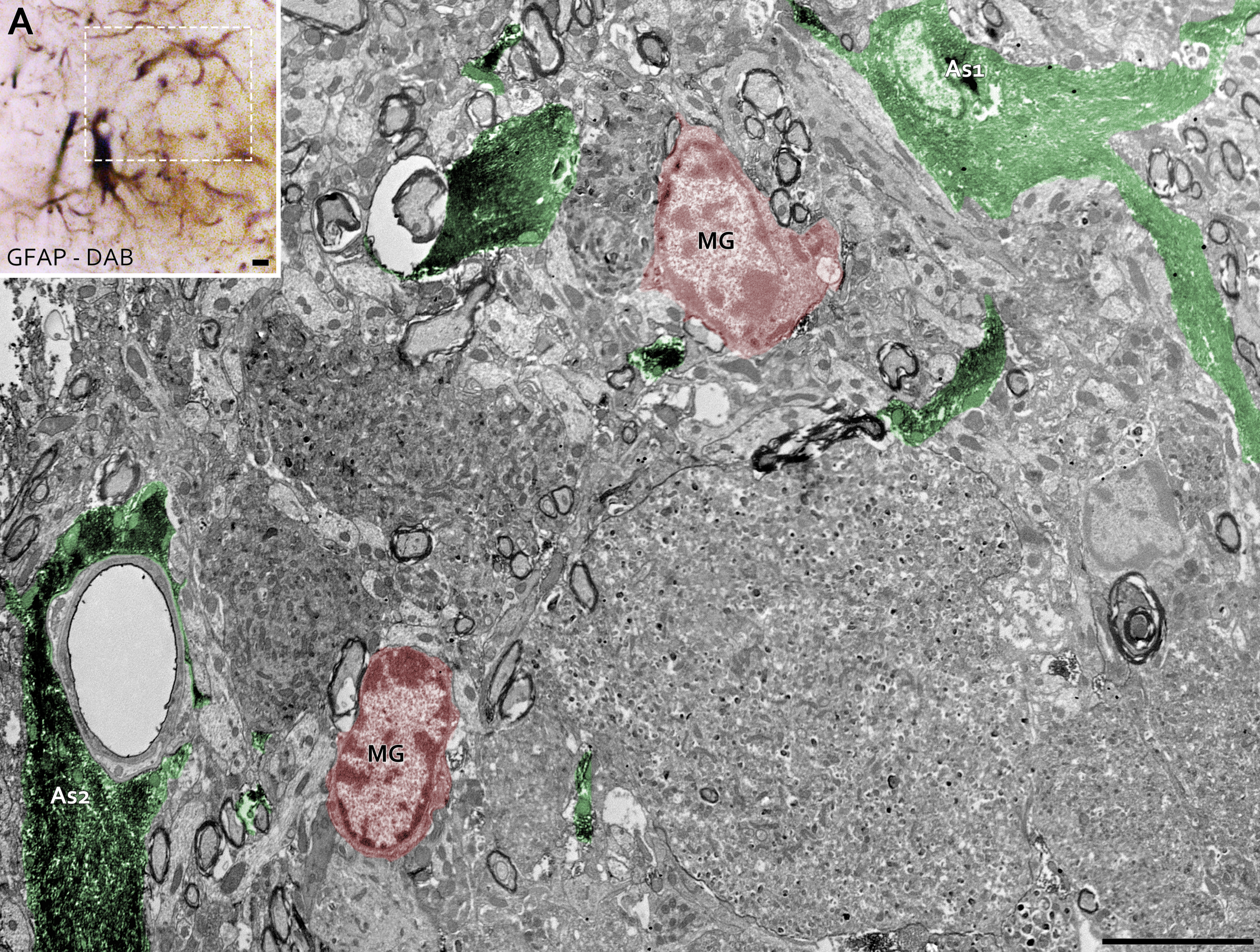

Supplement: S1 Fig — Representative correlated light (inset) and electron micrographs shows that an amyloid plaque is surrounded by microglia cells (MG, red) and GFAP-positive (DAB-labeled) astrocytes (green). Dystrophic neurites are located in the vicinity of the amyloid deposition bordered by astrocytes (As, green) which seem to separate damaged area from the environment. Scale bar: 4um. (TIF) [file pone.0233700.s001.tif]

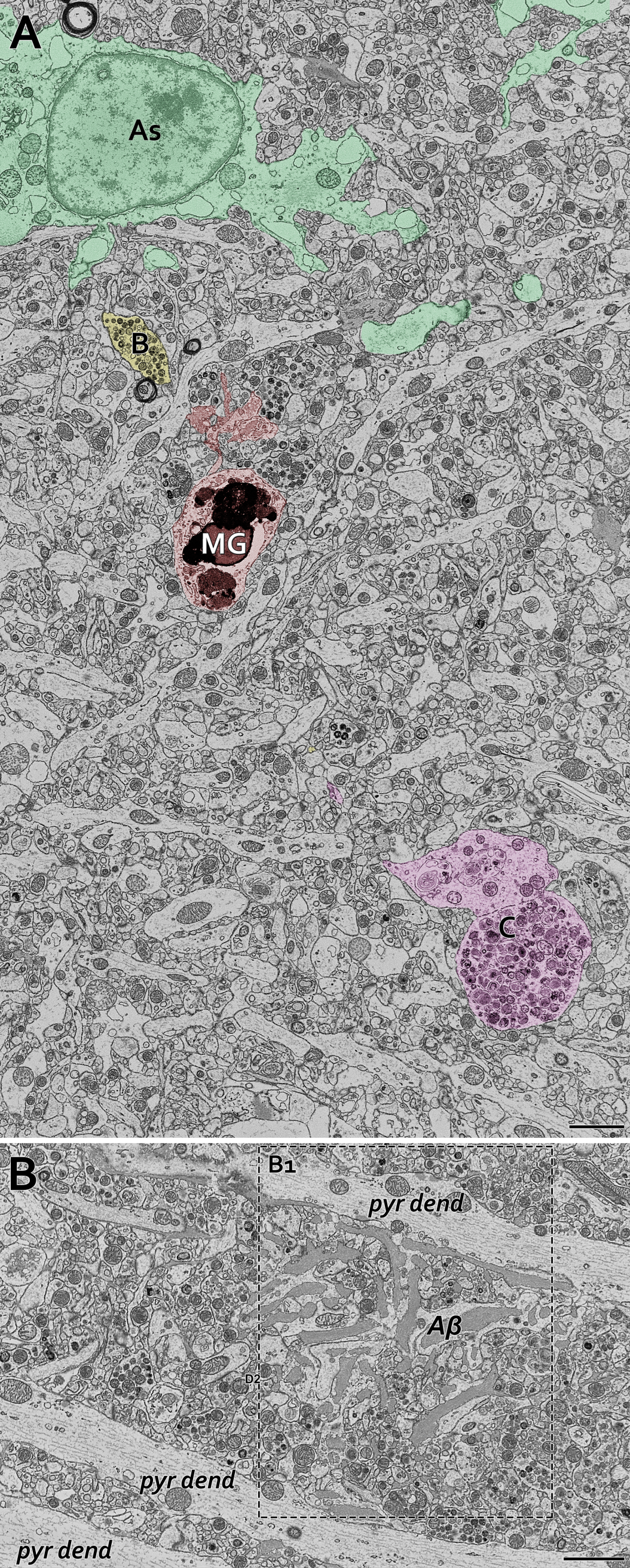

Supplement: S2 Fig — A: Representative section shows the environment of a mildly (B, yellow) and a strongly (C, pink) dystrophic axons described in Fig 2. (As: astrocyte, green; MG: microglia, red). B: Dendrites (pyr dend) of pyramidal cells in the stratum radiatum from hippocampus CA1. Aβ filaments build up around dendrites as a thin layer. Dashed area is presented in Fig 3C. Scale bar: 500 nm. (TIF) [file pone.0233700.s002.tif]
