# Supplementary material for: Amyloid β induces interneuron-specific changes in the hippocampus of APPNL-F mice
Source: PLoS One. 2020 May 29;15(5):e0233700. doi: 10.1371/journal.pone.0233700 (PMC7259556; doi:10.1371/journal.pone.0233700)
Supplement: S1 Table. Com — (DOCX) [file pone.0233700.s005.docx]

***Supplementary Table 1. Comparison of different anatomical properties of other three types of APP-expressing and APP^NL-F^ model mice***

| Animal model | APP/PS | 3xTg | 5xFAD | **APP^NL-F^** |
| --- | --- | --- | --- | --- |
| First presence of amyloid-β | Aβ deposition begins at **6 weeks** of age in the cortex and 3-4 months of age in the hippocampus ^1^. | Extracellular Aβ deposits by **6 months** in the frontal cortex ^2^ | Thioflavin-S-positive plaques emerge between **2 and 4 months** in frontal, parietal, entorhinal cortices and dentate gyrus ^3^. | We found first plaques appeared at **9 months** of age in several neocortical areas, then at around 12 months of age in the hippocampus. |
| Morphological alternations | Only dendritic spine loss around plaques reported to begin approximately 4 weeks after plaque formation ^4^. | The proportions of normal cells and cells with signs of damage were determined in the CA1 region, and damaged neurons were significantly more numerous in 3xTg-AD mice ^5^. | In 6-months-old mice the neuron numbers in hippocampal CA1 do not differ from wild-type mice ^6^.  Significant neuron loss occurs by 9 months of age ^7^. | In APPNL-F mice, we frequently observed cholinergic, glutamatergic and dopaminergic DNs but PV fibers are mostly spared form degeneration.  We found no difference in the density of SOM and PV INs; found no evidence of PNN deterioration. |
| GABA_A_  receptor  content | Amyloid deposits decreases the number of GABAergic perisomatic synapses on adjacent neurons ^8^.  In cerebral cortex reduction was observed in GABAergic innervation of AISs that were in contact with or inside the plaques ^9^. | In 7-month-old 3xTg alterations in the receptor subunits of GABA_A_ were studied ^10^.  No other anatomical data from studies. | No anatomical data. | We found no differece in the GABA_A_ receptor γ2 subunit positivity in practically all synapses established by PV positive somatic terminals, by PV positive axo-axonic INs terminals and by SOM IN terminals. |
| Synaptic areas | Expression of SYP and PSD95 were significantly reduced in the hippocampus ^11^. | Numeric density of synapses, average synaptic contact area, and synaptic surface density were investigated by morphometric methods in CA1 pyramidal cell layer. There was no statistically significant differences in any of the three parameters ^12^. | In electrophysiological studies synaptic deficits were detected in 8- to 12-week mice ^13^. | GABAergic synaptic clefts do not show significant differences.  Synapses on AISs were significantly larger in APP^NL-F^ mice by about 35%, but found no differences in somatic basket cell and SOM IN synaptic sizes. |
| Cognitive impairment | Cognitive deficits in spatial learning and memory in the Morris water maze reported at 7 months ^14^. | Cognitive impairment was detected at 4 months ^15,16^. | Impairments of spatial working memory, assessed in a cross-maze test, emerge between 3 and 6 months ^6^. | Natural anxiety is suppressed in APP^NL-F^ mice.  No alteration of spatial working and fear memory was detected. |
| 1. Radde, R. et al. *EMBO Rep.* **7**, 940–946 (2006).  2. Oddo, S. et al. *Neuron* **39**, 409–421 (2003).  3. Giannoni, P. et al. *Neurobiol. Dis.* **88**, 107–117 (2016).  4. Bittner, T. et al. *Acta Neuropathol.* **124**, 797–807 (2012).  5. Orta-Salazar, E., Feria-Velasco, A., Medina-Aguirre, G.I. & Díaz-Cintra, S. *Neurol. (English Ed.* **28**, 497–502 (2013).  6. Jawhar, S., Trawicka, A., Jenneckens, C., Bayer, T.A. & Wirths, O. *Neurobiol. Aging* **33**, 196.e29-196.e40 (2012).  7. Eimer, W.A. & Vassar, R. *Mol. Neurodegener.* **8**, 1 (2013).  8. Marin, M.A., J., Z., J., J. & M.N., R. *Exp. Neurol.* **281**, 93–98 (2016).  9. León-Espinosa, G., Defelipe, J. & Muoz, A. *J. Alzheimer’s Dis.* **29**, 841–852 (2012).  10. Revilla, S. et al. *Neuropharmacology* **81**, 55–63 (2014).  11. Jin, G. et al. *J. Physiol. Sci.* **69**, 477–488 (2019).  12. Bertoni-Freddari, C. et al. *Rejuvenation Res.* **11**, 309–313 (2008).  13. Buskila, Y., Crowe, S.E. & Ellis-Davies, G.C.R. *Neuroscience* **254**, 152–159 (2013).  14. Serneels, L. et al. *Science (80-. ).* **324**, 639–642 (2009).  15. Belfiore, R. et al. *Neuron* **324**, 409–421 (2012).  16. Billings, L.M., Oddo, S., Green, K.N., McGaugh, J.L. & LaFerla, F.M. *Neuron* **45**, 675–688 (2005). | | | | |
